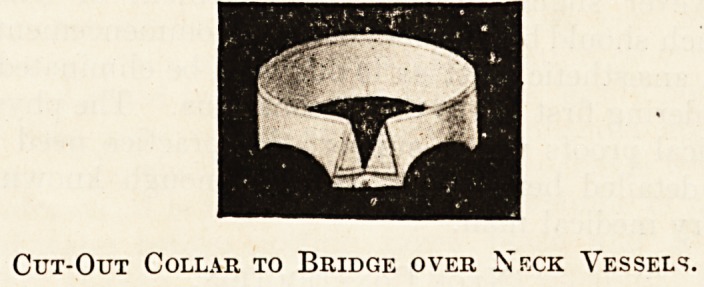# Cerebral Congestion and Tight Neck-Clothing

**Published:** 1911-05-27

**Authors:** Walter G. Walford


### The General Practitioner's Column.

[Contributions to this Column are invited, and, if accepted, will be paid for.]

### CEREBRAL CONGESTION AND TIGHT NECK-CLOTHING.

By WALTER G. WALFORD, M.D.

In all cases of fits, fainting, and unconsciousness generally where first aid is required, it is customary, as an initial step to all treatment, to unfasten the neck-clothes of the sufferer. So, too, the anæsthetist knows very well the importance of seeing to it that the patient has no tightly fitting neck-bands, for it is a well-known fact that any obstacle to the free circulation of the blood in the cerebral vessels, however slight, introduces an element of danger which should be eliminated at the commencement of the anæsthetic, just as it ought to be eliminated in rendering first aid to the unconscious. The physiological proofs which support this practice need not be detailed here; they are well enough known to every medical man.

### Neck Constriction.

But it is remarkable that no one seems to have noticed, or at any rate to have laid great stress on, the fact that such slight obstacles to the free circulation of the blood through the vessels of the brain may adversely influence the health of those who are in the habit of wearing tightly fitting neck-clothes. That fact, however, is well known to veterinary surgeons, who tell us that neck constriction through badly fitting harness is a causative factor in "staggers" in horses, and I have myself relieved a horse of this distressing symptom by relaxing the throat lash. It is equally well known that dogs suffer a great deal through having to wear tightly fitting collars. Is it too much, then, to argue that those of us who wear our own collars too tightly buttoned may find in that practice some explanation of symptoms ascribed to other causes?

### The Writer's Personal Experience.

Anyone who has seen many cases, suicidal or otherwise, of fatal neck constriction must have noticed three things: first, the small amount of actual constriction necessary to cause serious results; secondly, the fact that death in such cases is almost painless; and thirdly that such death cannot be caused by closure of the windpipe, as is so commonly supposed, except in rare instances, and then it cannot be painless. It is therefore reasonable to suppose that many of us must often unconsciously place ourselves within a danger zone, and that if we do this regularly we are bound to suffer for it in some way. The nerves and vessels of the neck are sufficiently close to the surface to suffer from continual pressure such as is exercised by tightly fitting collars. Since childhood the writer suffered from constant headaches, and with advancing years became rheumatic and gouty, with so-called "bilious attacks," the prominent symptoms of which were sudden vertigo and sickness. These attacks were regarded, quite erroneously, as was afterwards proved, as being due to congestion of the liver. The last attack occurred in March 1908, when the sickness lasted for forty hours, with pain in the head and diplopia, a very full, hard pulse, and a staggering gait. As the liver seemed healthy and the vessels of the fundus oculi were found to be congested, there was little doubt that this mischief was cerebral. A specialist confirmed this view, and added that there was probably a small thrombus in one of the cerebellar vessels. Though at first I improved a little, there was no marked amelioration in my condition, which in July 1908 was as follows: I had nearly constant pain in the head, especially when walking, with a feeling of dizziness: my pulse was still full and hard: I had old standing rheumatic gout in my knee, and more or less lumbago. Not at all a bright prospect at seventy years of age! I felt much better, usually, when I was completely undressed, and I soon became convinced that my illness was, at any rate, much influenced by my immediate surroundings. I therefore tried releasing my neck-clothes, and, finding that this considerably eased me, I had all my collars and neck-bands let out an additional inch. The result was a rapid improvement in my general condition that at the end of a fortnight I had resumed my bicycling. My head got better, my pulse became softer, and my gouty trouble left me, and has not, I am happy to say, returned since. I may say that I have thoroughly tested the influence of change of diet, but I now find that I can utterly disregard any modification of dietary so long as I stick to my enlarged collars. I found in addition that my neck was gradually enlarging, and I found that if I wished to keep pace with this enlargement I had to let out my collars further every six months. The explanation of my trouble appears to me to be very simple. From youth I had unconsciously, through wearing tight neck-clothing, prevented my neck from developing in proportion to the rest of my body. Directly I gave Nature a free hand, she made up the arrears, to the immense benefit of my health.

### Some Additional Cases.

The lesson I have thus learned through personal experience, I have tried to inculcate upon others. In a large number of patients, whom through my connection with an East End charity I was in the habit of seeing and whom I had hitherto treated for "constitutional conditions" which manifested themselves in gout and rheumatism, this simple plan of loosening the neck-clothing has worked wonders. Two instances will suffice, culled as they are from a large number of similar cases. One patient was a clergyman who suffered from constant headaches, with a tendency to get dazed and "lose himself," as he expressed it. When I saw him he had a very much flushed face and widely dilated pupils. On my recommendation he took a larger size in collars and all his bad symptoms left him, so that he is now in perfect health. Another patient, with similar symptoms, accompanied by vertigo and sickness, was in the habit of wearing a tight comforter which he fondly imagined was a prophylactic against catching cold. I had some difficulty in persuading him to give up this practice, but when finally my advice was followed, he improved rapidly and is now completely rid of his distressing symptoms.

A high Indian official had what was called an epileptic seizure, which was followed by profound constitutional symptoms. He was treated without any benefit, and finally advised to return home. His wife then, with great difficulty, induced him to follow the advice which I had given in my pamphlet on "Cerebral Congestion and Tight Neck-Clothing"—advice which, as he subsequently wrote to me, he considered "great rubbish!" To his astonishment he rapidly improved, and his resignation, which had already been handed in, was withdrawn. This gentleman is a complete stranger to me, but he now writes that he is in excellent health, and he ascribes his improved bodily condition entirely to the fact that he no longer wears tight neck-clothing. The neck is not a cylinder with parallel sides, but is more or less cone-shaped, an important difference which we often lose sight of. In investigating the condition of one's own collar one is apt to slip the fingers under it and raise to a level where the circumference of the neck is much smaller and where the difference between size of collar and the diameter of neck seems less marked. When the collar is allowed to slip back it naturally drops down into its ordinary working position where it presses on important and sensitive structures. The point to be remembered is that it is the lower edge of the collar that does the mischief, especially where it lies against the structures just above the clavicle. My own method of dealing with this compression is illustrated in the accompanying sketch. I use an ordinary collar of which the part shaded in the block has been cut out. By this method a kind of bridge is formed which is extremely comfortable, which enables the underlying parts to escape compression, and which can easily be hidden by the rim of the waistcoat aided by the ties.

One or two hints in conclusion. It is just as well not to relax the neck-clothing too suddenly. Also, never be content with merely relaxing the collar: see

Cut-Out Collar to Bridge over Neck Vessels.

that there is no constriction, however slight, at the upper end of the thorax, and that the shirt bands are never tightened, and pay particular attention to the collars of the patient's night-clothing. It is a little difficult to get patients to see that these hints are important. Prejudice, custom, and the erroneous idea about catching cold are hard to contend with. But it is worth while to insist and to see that your advice is carried out to the letter. The resultant benefits are too great and too striking to be disregarded.

## Figures and Tables

**Figure f1:**